# ^18^FDG uptake during induction chemoradiation for oesophageal cancer fails to predict histomorphological tumour response

**DOI:** 10.1038/sj.bjc.6603412

**Published:** 2006-10-03

**Authors:** C M Gillham, J A Lucey, M Keogan, G J Duffy, V Malik, A A Raouf, K O'Byrne, D Hollywood, C Muldoon, J V Reynolds

**Affiliations:** 1 Academic Unit of Clinical and Molecular Oncology, Trinity College Dublin, Trinity Centre, St James's Hospital, Dublin, Republic of Ireland; 2 Department of Nuclear Medicine/PET Centre, Trinity College Dublin, Blackrock Clinic, Blackrock Co., Dublin, Republic of Ireland; 3 Department of Radiology, Trinity College Dublin, St James's Hospital, Dublin, Republic of Ireland; 4 Department of Clinical Surgery, Trinity College Dublin, Trinity Centre, St James's Hospital, Dublin 8, Republic of Ireland; 5 Department of Pathology, Trinity College Dublin, St James's Hospital, Dublin, Republic of Ireland

**Keywords:** oesophageal cancer, positron emission tomography, neoadjuvant chemoradiation, response

## Abstract

To determine whether [^18^F]-fluorodeoxyglucose-positron emission tomography (FDG-PET) could predict the pathological response in oesophageal cancer after only the first week of neoadjuvant chemoradiation. Thirty-two patients with localised oesophageal cancer had a pretreatment PET scan and a repeat after the first week of chemoradiation. The change in mean maximum standardised uptake value (SUV) and volume of metabolically active tissue (MTV) was compared with the tumour regression grade (TRG) in the final histology. Those who achieved a TRG of 1 and 2 were deemed responders and 3–5 nonresponders. In the responders (28%), the SUV fell from 12.6 (±6.3) to 8.1 (±2.9) after 1 week of chemoradiation (*P*=0.070). In nonresponders (72%), the results were 9.7 (±5.4) and 7.1 (±3.8), respectively (*P*=0.003). The MTV in responders fell from 36.6 (±22.7) to 22.3 (±10.4)  cm^3^ (*P*=0.180), while in nonresponders, this fell from 35.9 (±36.7) to 31.9 (±52.7) cm^3^ (*P*=0.405). There were no significant differences between responders and nonresponders. The hypothesis that early repeat FDG-PET scanning may predict histomorphologic response was not proven. This may reflect an inflammatory effect of radiation that obscures tumour-specific metabolic changes at this time. This assessment may have limited application in predicting response to multimodal regimens for oesophageal cancer.

The standard therapy for localised oesophageal carcinoma is surgical resection (Enzinger and Mayer, 2003). However, local control and overall survival remain poor, and even after radical resection and lymphadenectomy, the 5-year survival is at best approximately 40% (Lerut *et al*, 1999; Altorki *et al*, 2002). In an effort to improve outcomes, neoadjuvant approaches, either chemotherapy alone or combined with radiation therapy, have been evaluated in randomised clinical trials (Nygaard *et al*, 1992; Le Prise *et al*, 1994; Walsh *et al*, 1996; Bosset *et al*, 1997; Kelsen *et al*, 1998; Urba *et al*, 2001; Medical Research Council Oesophageal Cancer Working Group, 2002; Burmeister *et al*, 2005). The sole prospective randomised trial to report survival benefit for neoadjuvant combination chemotherapy and radiation therapy compared with surgery alone was undertaken at this centre between 1990 and 1995 (Walsh *et al*, 1996). Although controversial, the use of this multimodality approach has increased outside of clinical trials, and the patterns of care studies in the US showed that preoperative chemoradiation therapy increased from 10.4% during 1992–1994 to 26.6% in 1996–1999 (Suntharalingam *et al*, 1999).

The patients who receive maximum benefit from neoadjuvant combination chemotherapy and radiation therapy (CRT) are those who achieve a complete pathological response (pCR), with no residual cancer cells in the primary tumour or lymph nodes. A pCR occurs in approximately 15–30% of cases, and 3-year survival rates of approximately 60% irrespective of the applied protocol, type of histology and tumour stage are achieved (Geh *et al*, 2001). A further subdivision of pathological response to neoadjuvant regimens, the tumour regression grade (TRG) (Mandard *et al*, 1994), may also identify patterns of incomplete response that may impact on treatment outcome, and the addition of the pathologic response to pTNM staging has been recently advocated (Swisher *et al*, 2005). Where a cohort of patients may benefit from neoadjuvant CRT, with pCR and TRG the surrogate markers, many patients will not be helped, and their prognosis may be worsened by delay in surgery and by the added risks of surgery in patients on multimodal protocols (Ancona *et al*, 2001; Fiorica *et al*, 2004). A predictor of response or resistance would have potentially enormous application in optimising outcomes. Moreover, new markers that function as surrogate or proxy indicators of histomorphological response may be of great value in the design of Phase II studies using new treatment regimens including molecular therapies. At this time, pretreatment demographics, cross-sectional imaging, histopathologic, molecular or genetic information have not been of clinical value as early response predictors.

An alternative approach would be identification of a metabolic pattern that may emerge early after the induction of treatment, which might then guide management. [^18^F]-fluorodeoxyglucose-positron emission tomography (FDG PET) scanning may be such a predictive tool. It is already established in the staging of oesophageal cancer (Kneist *et al*, 2003), and recent evidence suggests early response patterns to neoadjuvant chemotherapy that determine outcome (Downey *et al*, 2003; Wieder *et al*, 2004). The earliest time point identifying response or resistance will have the greatest potential application, and in this study we tested this hypothesis after a minimum of 7 days of combination chemoradiation. We report herein FDG uptake, using both standardised uptake value (SUV) and assessment of metabolically active tumour volume (MTV), that early patterns were not evident that predicted histomorphologic response.

## PATIENTS AND METHODS

Between January 2003 and October 2005, 32 consecutive patients with histologically confirmed oesophageal carcinoma scheduled to undergo multimodal therapy underwent an FDG-PET scan at diagnosis and following the first week of CRT. Each patient gave informed consent. Pretreatment investigations also included computerised tomography of the neck, thorax and abdomen, and oesophagogastroscopy. The criteria for inclusion in the multimodal protocol was as follows: age <77; satisfactory performance status and medical fitness for surgery; a biopsy-proven tumour of the oesophagus or oesophagogastric junction; and a staged tumour deemed resectable by the primary surgeon. All patients had, in addition, a leucocyte count greater than 3500 cm^3^, a platelet count above 100 000 cm^3^, serum creatinine less than 1.4 mg dl^−1^ (124 *μ*mol l^−1^), no previous chemotherapy or radiation therapy and no previous cancer other than that of the skin.

The treatment protocol was as described previously (Walsh *et al*, 1996), with 3–4 weeks of radiation therapy, the first combined with chemotherapy, further chemotherapy alone in week 5 and surgery approximately 1 month later. For radiotherapy, the planning target volume incorporated the gross tumour volume plus a 4–5 cm margin superiorly and inferiorly with 2 cm circumferentially. This was identified using the information gained from the endoscopy and diagnostic CT scans, as well as by the use of a barium swallow during simulation. The dose of radiotherapy was either 40.05 Gy in 15 daily fractions over 3 weeks or 44 Gy in 22 daily fractions over four and a half weeks. Each dose was prescribed to the mid-plane using 10–15 MV photons. 5-Fluorouracil at 15 mg kg^−1^ was delivered on days 1–5 and cisplatin at 75 mg m^−2^ on day 6. Surgery involved transthoracic oesophagectomy including *en bloc* lymphadenectomy of the abdominal and mediastinal nodes, and it was not undertaken until the neutrophil count was consistently above 2000 *μ*l^−1^ on three successive occasions in a 2-week period.

### FDG-PET imaging

[^18^F]-fluorodeoxyglucose-positron emission tomography scans were performed on all patients as part of their staging and in the week following completion of the induction combination chemotherapy and radiation therapy. The PET images were acquired on a high-resolution dedicated PET scanner 47–78 min after intravenous injection of 340–450 MBq of fluorine-18-flurodeoxyglucose (^18^F-FDG). In so far as was possible, the scanning conditions were kept constant facilitating comparison of the pretreatment and intratreatment scans, that is, same acquisition protocol, reconstruction algorithm and uptake time (mean Δ*t*=3.9 min). Patients fasted for 6 h before imaging to ensure that serum glucose and endogenous serum insulin levels were low at the time of FDG administration. Blood glucose levels were measured before each FDG-PET scan. Whole-body scans extending from base of skull to mid-thigh were obtained in 2-D mode on either the GE-supplied *PET Advance* scanner or *Discovery-ST* PET/CT scanner. The early repeat image was always performed on the same scanner as the initial. The images were reconstructed using ordered subsets expectation maximum iterative reconstruction.

Semiquantitative measurements of metabolic uptake in FDG-avid tumours following pretreatment and intratreatment scans were compared and evaluated for their potential to predict histopathological response to CRT. The tumour FDG uptake was measured using a region of interest (ROI) method (Stahl *et al*, 2004). Briefly, a cylindrical ROI with a diameter of 1.5 cm was manually placed over the tumour site on the hottest trans-axial slice, avoiding the edges of the tumour. The mean activity concentration within the ROI was determined and expressed as the SUV, where SUV is the ratio of the activity in the tissue to the decay-corrected activity injected into the patient. This technique combines the advantages of little interference by statistical count rate fluctuations and little influence by nonviable tumour zones or partial volume effects. The other parameter we studied was the volume of metabolically active tissue (MTV) disease. This was selected by choosing a threshold SUV value, in which only voxels with SUV values greater than or equal to the selected threshold were included in the volume. All SUV measurements were normalised for patient body weight (SUV). The relative changes in tumour SUVs between baseline and follow-up were calculated and correlated with subsequent histopathological tumour response to therapy.

The percentage change (Δ) in each of the parameters (*P*) between diagnosis (pre) and during treatment (intra) was calculated using the following formula:

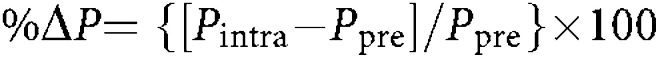

A negative value indicated a reduction in that parameter following therapy and a positive value indicated an increase.

### Histology

All the surgical specimens were classified by one experienced pathologist (CM) who was unaware of the clinical and PET data and who graded and staged the specimens in accordance with the criteria of the International Union Against Cancer and the American Joint Committee on Cancer Staging (Greene *et al*, 2002). Tumour response to treatment was classified according to the criteria described by Mandard *et al* (1994). Complete response showed histologic fibrosis with or without inflammation extending through the different layers of the oesophageal wall, but with no viable residual tumour cells (TRG 1). Subtotal response (TRG 2) was characterised by the presence of rare residual cancer cells scattered through the fibrosis. An increase in the number of residual cancer cells, but with fibrosis predominating, was termed a partial response (TRG 3). Minimal response (TRG 4) showed residual cancer outgrowing fibrosis. The absence of any regressive changes (TRG 5) defined no change.

### Statistical Analysis

A statistical analysis was performed using commercial software SPSS for Windows (version 12.0). Intraindividual comparisons were performed using the Wilcoxon signed-rank test and interindividual comparisons using Wilcoxon/Kruskal–Wallis. The data were analysed in two ways: first designating a TRG of 1 and 2 as responders with the rest nonresponders and second designating TRG of 1–3 as responders in keeping with the original Mandard paper (Mandard *et al*, 1994).

## RESULTS

The basic demographics are shown in Table 1. The median age was 59 years, there was a male preponderance, and a majority of patients had adenocarcinoma. Most patients had clinical Stage 2 disease.

### TRG 1 and 2 *vs* 3–5

**Figure 1:**
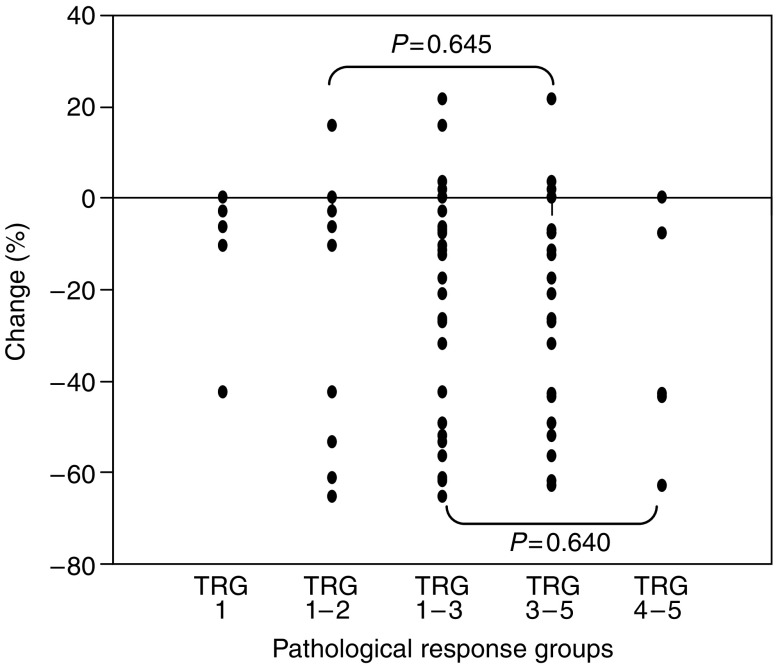
Percentage change in mean maximum SUV from pre- to during CRT relative to differing pathological response groups. TRG, tumour regression grade.

**Figure 2:**
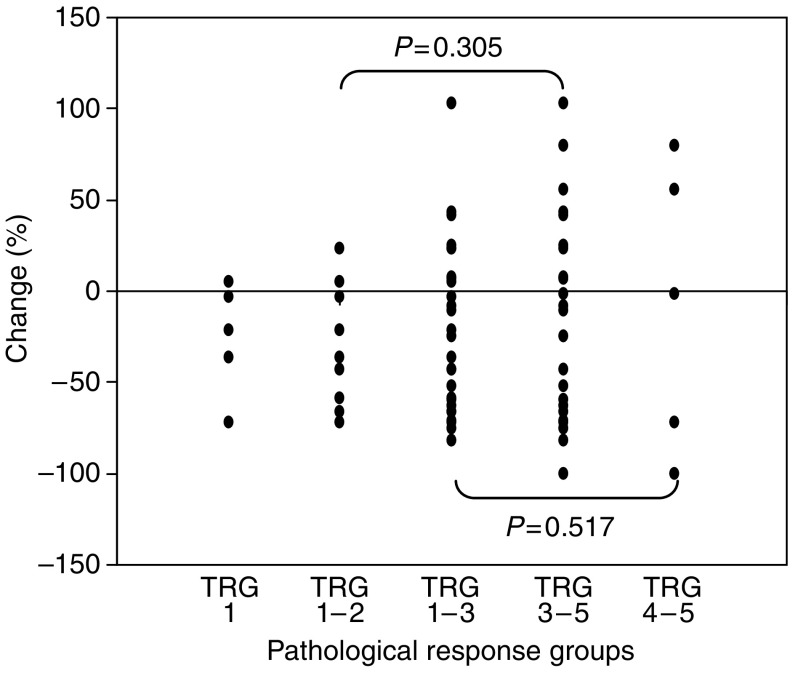
Percentage MTV from pre- to during CRT relative to differing pathological response groups. TRG, tumour regression grade.

(Table 2 and Figures 1 and 2) Of the 32 patients, nine (28%) achieved a total or near total response (TRG 1 or 2) and 23 (72%) had less or no response (TRG 3, 4 or 5). The 1-year survival in the TRG 1 and 2 group was 87% compared with 67% in TRG 3–5 group (*P*=0.091). There was no significant correlation between either the initial SUV (*P*=0.191) or MTV (*P*=0.472) scores and the final pathological response.

In the responder group 9 of 32, the mean (s.d.) SUV fell from 12.6 (6.3) g ml^−1^ pretreatment to 8.1 (2.9) g ml^−1^ following 1 week of chemoradiation (*P*=0.070), while in nonresponders (23 of 32), it fell from 9.7 (5.4) to 7.1 (3.8) g ml^−1^, respectively (*P*=0.003). The MTV in good responders fell from 36.6 (22.7) to 22.3 (10.4) cm^3^ during treatment (*P*=0.180), while in nonresponders, this fell from 35.9 (36.7) to 31.9 (52.7) cm^3^ (*P*=0.405). There was a mean reduction in SUV of 25.2 and 22.3% in responders and nonresponders, respectively (*P*=0.902). There was a mean reduction in MTV of 30.4 and 15.1% in responders and nonresponders, respectively (*P*=0.621). The change in SUV pretreatment and after induction CRT was not significantly different between responders and nonresponders (*P*=0.645). Similarly, the change in MTV pretreatment and after induction CRT was not significantly different between responders and nonresponders (*P*=0.305).

In an attempt to identify a threshold above or below which response could be more accurately predicted, a reduction of more than 20% in each of the parameters was used as a criterion. The positive predictive values were 27 and 35% for changes in SUV and MTV, respectively. The negative predictive values were 71 and 80%, respectively for the same parameters. No other cutoff value was found to differentiate responding from nonresponding tumours better.

### TRG 1–3 *vs* TRG 4 and 5

(Table 2 and Figures 1 and 2) In this analysis, there were 27 patients in the TRG 1–3 group (responder) and five patients in the TRG 4 and 5 group (nonresponder). There was no significant correlation between either the initial SUV (*P*=0.324) or MTV (*P*=0.483) scores and the final pathological response. In the responder group, the mean (s.d.) maximum SUV fell from 10.6 (5.7) g ml^−1^ pretreatment to 7.5 (3.5) g ml^−1^ following 1 week (*P*=0.002). In nonresponders, the results were 10.1 (6.3) and 6.7 (4.2) g ml^−1^, respectively (*P*=0.125). The MTV in responders fell from 32.7 (26.8) to 22.3 (24.3) cm^3^ during treatment (*P*=0.124), while in nonresponders, this rose from 54.5(57.2) to 66.0 (99.1) cm^3^ (*P*=0.893). The change in SUV pretreatment and after induction CRT was not significantly different between responders and nonresponders (*P*=0.640). Similarly, the change in MTV pretreatment and after induction CRT was not significantly different between responders and nonresponders (*P*=0.517). There was a mean reduction in SUV of 21.6 and of 31.4% in responders and nonresponders, respectively (*P*=0.479). There was a mean reduction in MTV of 21.6 and 7.8% in responders and nonresponders, respectively (*P*=0.841). Using a greater than 20% threshold, the positive predictive values were 80 and 88% for changes in mean maximum SUV and MTV, respectively.

## DISCUSSION

In contrast to endoscopic ultrasound and CT imaging, which cannot differentiate fibrous tissue from viable tumour tissue in patients undergoing neoadjuvant treatment regimens, FDG-PET scanning holds greater promise. It monitors glucose turnover in glucose-avid tumour cells, and, intuitively, diminished metabolism should be anticipated to correlate with a tumour response. Moreover, the metabolic changes may precede structural changes, and this has been confirmed for certain solid tumours (Smith, 1998). The advent of PET/CT has enhanced the anatomic localisation of lung tumours (Lardinois *et al*, 2003). To our knowledge, there have not been equivalent studies in oesophageal cancer. However, the recording of SUV or MTV would not have been altered by the addition or absence of the CT component.

In studies in oesophageal cancer, the FDG-PET scan has predominantly been performed following completion of the neoadjuvant protocol, as distinct from early in the treatment (Brücher *et al*, 2001; Arslan *et al*, 2002; Flamen *et al*, 2002; Kato *et al*, 2002; Downey *et al*, 2003; Brink *et al*, 2004; Wieder *et al*, 2004). The evidence from these studies suggests that FDG-PET scans following treatment significantly correlates with pathologic response and survival. Brücher *et al* (2001) prospectively evaluated 37 patients following chemoradiation for T2–4 squamous cell tumours, of whom 24 proceeded to surgery. [^18^F]-fluorodeoxyglucose-PET was performed at the completion of chemoradiation, and responders were defined as less than 10% viable tumour cells, correlating closely with TRG 1 and 2 in this study, FDG uptake decreased by 72±11%, while in nonresponders, the decrease was significantly (*P*=0.002) less at 42±22%, and this was associated with worse overall survival. Brink *et al* (2004) prospectively studied 20 consecutive patients who underwent surgery following chemoradiation. Although the SUV decreased (*P*<0.01) in all patients following the neoadjuvant component, the percentage change did not differ significantly between responders and nonresponders.

An early marker of response offers the greatest potential clinical advantage, particularly if those not benefiting from treatment could be identified and offered alternative approaches, and this was the hypothesis evaluated in this study. In a similar study, but using chemotherapy alone, Weber *et al* (2001) performed the second PET scan in 40 consecutive patients just before the second of two cycles of chemotherapy. A significant difference in tumour FDG uptake between responders and nonresponders was observed and, applying a cutoff value of 35% reduction of initial FDG uptake as a criterion for metabolic response, they were able to predict clinical response with 93% sensitivity and 95% specificity. Where early sequential chemoradiation was employed, the most comparable study is from Wieder *et al* (2004), who performed the second PET scan following 2 weeks of neoadjuvant chemoradiation in 27 patients with oesophageal squamous cell cancer. Mean tumour SUV in the group was 9.3±2.8 g ml^−1^ before therapy and decreased to 5.7±1.9 g ml^−1^ (−38±18%; *P*<0.001) after 2 weeks. In histopathologic responders, equivalent to TRG 1 and TRG 2, the decrease in SUV from baseline to day 14 was 44±15%, whereas it was only 21±14% in nonresponders (*P*=0.005). Although in the present series both the maximum SUV and MTV decreased more in responders compared with nonresponders, this does not approach significance. There are some differences in this study and that of the Munich group (Wieder *et al*, 2004): the report by the Munich group was in squamous cell cancer, whereas this study was predominantly in patients with adenocarcinoma; the second week of induction CRT was completed in the Munich study before the second FDG-PET scan was performed, in contrast to this study where the second scan was performed after 1 week of induction therapy.

It is likely that the inflammatory response to radiation obscures changes in tumour glucose metabolism associated with treatment effect (Hautzel and Muller-Gartner, 1997). This effect would be expected in both responders and nonresponders and might mask a reduction in SUV. It is difficult to suggest that this would be any different at 2 weeks. Although it is possible that larger sample sizes may help discriminate responders and nonresponder, the observation that a decrease in SUV and MTV is frequently observed in patients who do not achieve a major histomorphologic response at least suggests that early sequential scans in patients receiving radiation as part of their induction regimen is unlikely to be a good discriminant of response or resistance.

In conclusion, the hypothesis that histological tumour responses at surgery after induction CRT may be identified by early FDG-PET scans has not been proven. These results appear to contradict previously reported findings (Weber *et al*, 2001, 2004), but an early inflammatory response to radiation even at 7 days may be the confounding variable. Larger studies are required, but response evaluation using metabolic imaging might be easier to evaluate in trials of induction chemotherapy alone or new biological agents, before the administration of radiation therapy.

## Figures and Tables

**Table 1:** Patient characteristics

Male/female	34/7
Mean age (range) (years)	58 (37–74)
	
*Pathology*	
Squamous cell	5
Adenocarcinoma	27
	
*Grade*	
Well differentiated	1
Moderately differentiated	16
Poorly differentiated	13
Not evaluable	2
	
*Primary site*	
Middle	4
Lower	16
O–G junction	12
	
*Clinical stage*	
II	27
III	5
	
*Radiotherapy dose*	
44 Gy in 22 fractions	11
40.05 Gy in 15 fractions	21
	
*Fractions of RT completed before second PET*	
Mean	7
Range	5–10

CRT=chemoradiation; Gy=Gray; O–G=oesophagogastric; PET=positron emission tomography; RT=radiotherapy.

**Table 2:** Evaluable patients – clinical stage, pathological response and FDG uptake

**Patient**	**Pre-CRT clinical stage**	**TRG**	**Pathological stage**	**SUV pre (g ml^−1^)**	**SUV intra (g ml^−1^)**	**Reduction in SUV (%)**	**MTV pre (g ml^−1^)**	**MTV intra (g ml^−1^)**	**Reduction in MTV (%)**
1	T3N0M0	1	ypT0N0	6.3	5.9	6.4	24.6	19.2	21.9
2	T3N0M0	1	ypTxN0	6.1	3.5	42.6	13.4	3.7	72.1
3	T3N1M0	1	ypT0N0	12.8	11.5	10.4	35.6	34.5	3.2
4	T3N0M0	1	ypT0N0	12.8	12.8	0.0	35.3	22.4	36.5
5	T3N0M0	1	ypT0N0	9.8	9.5	3.0	19.6	20.6	−5.0
6	T3N0M1a	2	ypT3N0	5.1	5.9	−15.7	13.2	16.2	−23.2
7	T3N0M0	2	ypT1N0	19.2	8.9	53.4	80.0	33.0	58.8
8	T3N0M0	2	ypT3N0	21.4	8.3	61.2	61.6	35.3	42.7
9	T3N0M0	2	ypT1N0	19.6	6.8	65.3	46.0	15.5	66.7
10	T3N0M0	3	ypT3N1	19.7	8.6	56.4	48.5	13.7	71.7
11	T3N1M0	3	ypT3N1	10.0	4.8	52.0	25.4	4.7	82.0
12	T3N0M0	3	ypT3N0	9.5	7.0	26.3	25.4	10.1	60.1
13	T3N0M0	3	ypT3N1	20.9	18.3	12.4	95.9	87.6	8.7
14	T3N1M0	3	ypT2N1	12.4	4.7	62.1	22.3	5.5	75.4
15	T3N1M0	3	ypT2N0	12.6	11.7	7.0	4.6	5.8	−25.3
16	T3N0M0	3	ypT3N1	6.4	5.9	7.8	10.2	5.8	43.0
17	T3N0M0	3	ypT3N0	6.0	7.3	−21.7	17.7	13.3	24.9
18	T3N0M0	3	ypT3N0	5.7	4.5	21.1	22.9	28.3	−23.4
19	T2N1M0	3	ypT3N1	9.3	4.7	49.5	46.6	15.9	66.0
20	T3N0M0	3	ypT3N1	4.3	3.8	11.6	7.9	7.1	10.8
21	T3N0M0	3	ypT2N1	5.6	5.7	−1.8	11.6	23.6	−102.5
22	T3N0M0	3	ypT3N0	8.2	8.5	−3.7	21.3	22.6	−6.2
23	T3N0M0	3	ypT3N1	4.4	3.2	27.3	26.0	12.4	52.3
24	T3N0M0	3	ypT3N0	19.1	13.0	31.9	53.3	19.6	63.2
25	T3N0M0	3	ypT3N1	6.2	5.1	17.7	7.6	10.9	−43.3
26	T3N0M0	3	ypT2N1	4.3	4.3	0.0	3.5	4.9	−41.2
27	T3N0M0	3	ypT3N1	8.7	8.7	0.0	103.7	111.3	−7.3
28	T3N0M0	4	ypT3N0	6.2	3.5	43.6	16.9	0.0	100.0
29	T3N0M0	4	ypT4N1	20.8	11.9	42.8	154.0	239.0	−55.2
30	T3N0M0	4	ypT3N1	10.5	10.5	0.0	32.4	58.2	−79.6
31	T3N1M0	4	ypT3N0	7.8	2.9	62.8	49.8	13.9	72.1
32	T3N0M0	4	ypT3N1	5.2	4.8	7.7	19.2	18.9	1.7

CRT=chemoradiation; FDG=fluorodeoxyglucose; intra=during CRT; MTV=metabolic tumour volume; pre=before CRT; SUV=mean maximum standardised uptake value; TRG=tumour regression grade.
